# Exploring the relationship between clinical symptoms and MRI findings in temporomandibular joint disorders: a preliminary study

**DOI:** 10.22514/jofph.2025.059

**Published:** 2025-09-12

**Authors:** Ecem Sancar, Dilek Yılmaz, Safak Parlak, Elif G. Bulut, Gozde Ozer, Nur E. Hersek

**Affiliations:** ^1^Department of Prosthodontics, Faculty of Dentistry, Baskent University, 06490 Ankara, Turkey; ^2^Department of Oral Radiology, Faculty of Dentistry, Baskent University, 06490 Ankara, Turkey; ^3^Department of Radiology, Faculty of Medicine, Hacettepe University, 06230 Ankara, Turkey; ^4^Department of Prosthodontics, Faculty of Dentistry, Hacettepe University, 06230 Ankara, Turkey

**Keywords:** Temporomandibular joint disorder (TMD), Magnetic resonance imaging (MRI), Joint effusion, Disc deformity

## Abstract

**Background**: This study aimed to evaluate the relationships between magnetic resonance imaging (MRI) findings (such as condylar degeneration, disc displacement, joint effusion and disc deformity) and clinical symptoms in patients with temporomandibular joint disorders (TMDs). **Methods**: A total of 54 patients (108 temporomandibular joints (TMJs)) were included. Clinical evaluations assessed joint pain, joint sounds, mouth opening limitations, deviation/deflection and locking. MRI scans were analyzed for condylar degeneration, disc displacement (disc displacement with reduction (DDWR) or disc displacement without reduction (DDWOR)), joint effusion, and disc morphology. Statistical analyses included chi-square tests/Fisher’s exact tests for categorical variables. A *p* value < 0.05 was considered to be statistically significant. **Results**: DDWR and DDWOR were significantly associated with joint pain (*p* = 0.044) and sounds (*p* = 0.032). Joint effusion demonstrated no clear correlation with clinical symptoms. Condylar degeneration was frequently observed but had limited clinical impact, except for a reduction in joint sounds (*p* = 0.03). Moreover, disc deformity was significantly correlated with condylar degeneration and joint effusion (*p* < 0.001). **Conclusions**: MRI findings provide valuable insights into the structural changes observed in TMDs. Although disc displacement is strongly linked to pain and joint sounds, condylar degeneration and effusion exhibit more complex relationships with clinical symptoms.

## 1. Introduction

Temporomandibular joint disorders (TMDs) represent a group of musculoskeletal conditions that affect the temporomandibular joint (TMJ) and its associated structures, including the masticatory muscles and the articular disc. TMDs are characterized by clinical signs and symptoms, including pain in the TMJ and face, joint noise and irregular jaw movements [1, 2].

Condylar degeneration is characterized by wear, irregularity, and structural deterioration of the joint surface [3, 4]. This degeneration often develops due to various factors, such as osteoarthritis, trauma, malocclusion, bruxism, or inflammatory diseases [5]. Clinical findings may include pain and tension in the TMJ, restricted mouth opening, and frequent clicking or popping sounds. In more advanced stages, crepitus may also be detected [6, 7, 8].

Anterior disc displacement with reduction (DDWR) and without reduction (DDWOR) are the most common disorders of the TMJ [9]. This condition is characterized by the displacement of the articular disc from its normal position, which disrupts the alignment between the mandibular condyle and the temporal bone [10].

In the early stages of the disorder, the disc exhibits anterior DDWR, which is typically accompanied by a “clicking” sound in the joint [11]. As the condition progresses, the joint structures become stretched, and the disc is no longer able to return to its original position, thereby leading to DDWOR [10, 12]. In the initial stages of DDWOR, mouth opening is limited, and deviation toward the affected side may be observed. However, in later stages, the mouth opening capacity increases. This process can ultimately lead to degenerative changes in the TMJ [12, 13, 14].

One of the most investigated aspects of TMDs is the presence of joint effusion, which refers to the accumulation of synovial fluid within the TMJ space and is commonly detected using MRI [15, 16]. Effusion is often considered an indicator of intra-articular inflammation and has been associated with conditions such as internal derangement and osteoarthritis [17, 18, 19].

Deformation of the disc in the TMJ is characterized by the loss of its normal anatomical structure and subsequent deformation. Deformities of disc configuration are associated with internal derangement of the TMJ and condylar degenerative changes [12, 14].

Magnetic resonance imaging (MRI) is a widely used technique and is considered the gold standard for evaluating TMDs [20, 21]. This technique allows for a detailed assessment of condylar degeneration, disc position, the presence of effusion, and disc morphology [22].

The aim of this study was to evaluate the relationships between parameters such as condylar degeneration, disc displacement, the presence of effusion, and disc deformity detected in MR images and clinical findings in patients with intra-articular disorders.

## 2. Materials and methods

This study was conducted with data obtained from patients who presented with TMJ complaints to the Department of Prosthodontics, Faculty of Dentistry, Hacettepe University, between October 2019 and March 2020. The TMJ findings of 54 patients (48 females and 6 males) with a mean age of 29.63 ± 3.90 years (range: 15–66 years) were evaluated. Patients presenting with complaints of discomfort in the TMJ region and suspected of having internal derangement were clinically evaluated for TMD. All the patients were examined by a single clinician, and an examination form that was prepared to assess clinical symptoms was thoroughly completed for each patient. This study was approved by the Ethics Committee of Hacettepe University (No: GO19/536), and informed consent was obtained from all the patients.

### 2.1 Inclusion criteria

1. The presence of joint sounds during mouth opening and closing (either unilaterally or bilaterally), which could be reproduced in at least two out of three consecutive trials and could disappear upon protrusive opening, or a history of joint sounds.

2. Restricted mouth opening accompanied by deviation toward the affected side, a history of jaw locking, or episodes of locking and catching sensations.

3. Pain in the jaw, face or within the ear, along with tenderness upon palpation.

4. Pain in one or both TMJs upon palpation, as well as pain experienced during joint function.

### 2.2 Exclusion criteria

1. Patients with general contraindications to MRI, such as those with cardiac pacemakers or other metallic implants incompatible with MRI.

2. Patients with claustrophobia leading to premature termination of the examination.

3. Patients with clinical signs and symptoms indicative of TMJ muscle disorders or a history of prior treatment for such conditions.

### 2.3 Clinical examination

The clinical examination consisted of evaluations of TMJ pain, TMJ noise and maximum mouth opening, and clinical diagnoses were made according to the Diagnostic Criteria for Temporomandibular Disorders (DC/TMD) [23, 24].

Pain in the TMJ was assessed using the visual analog scale (VAS). In this scale, patients were asked to rate their pain on a scale from 0 to 10, where 0 represents no pain and 10 represents unbearable pain. Patients marked their pain levels on the scale to objectively indicate the intensity and severity of the pain in the TMJ. This method provides a standardized measurement of pain, which is used to evaluate the progress of the treatment process.

To determine the presence of sounds in the temporomandibular joint, the diaphragm of a stethoscope was placed over the joint area. Patients were asked to open and close their mouths, and sounds emitted from the joint were carefully monitored during these movements. To ensure that the sounds were clearly audible, the patient was asked to repeat the mouth-opening motion three times, and the sounds detected from the joint were recorded. This method helps in identifying any sounds that could indicate abnormalities or pathological conditions in the joint.

To assess maximum mouth opening, patients were asked to open their mouths as wide as possible three times, and the greatest degree of opening was recorded. The maximum opening was measured by placing a caliper between the incisal edges of the upper and lower central incisors, and the measurement was recorded in millimeters using a ruler. This method allows for an objective and standardized determination of mouth opening.

Deviations occurring during mouth opening were assessed via visual observations. Patients were asked to close their mouths in centric occlusion and then slowly open their mouths. This movement was repeated several times to observe whether any deviation occurred.

A history of joint locking in the TMJ was assessed based on the patients’ previous complaints and symptoms related to the joint. Patients were asked whether they had experienced any episodes of catching, locking or sudden restrictions of movement and whether such events had occurred in the past. The sensation of locking was defined as the sudden restriction of joint mobility or difficulty with opening the mouth due to pain, and the presence of this symptom was considered an important indicator potentially related to temporomandibular dysfunction.

### 2.4 MRI technique

All of the MR images were obtained from a 1.5 T scanner (Achieva, Philips Healthcare, Best, The Netherlands).

The protocol for TMJ MRI was the same for all of the MRI examinations in the sagittal oblique plane, short tau inversion recovery (STIR) (repetition time (TR)/echo time (TE)/inversion time (TI): 2374/60/180 ms, flip angle (FA): 90°, slice thickness (ST): 3 mm, number of excitations (NEX): 2, field of view (FOV): 150 × 150) and T1-weighted (W) 3D WATS (water-selective sequence) (TR/TE: 30/5 ms, FA: 30°, ST: 2 mm, NEX: 2, FOV: 150 × 150) for the closed-mouth position, as well as proton density weighted (PDW) turbospin echo (TSE) (TR/TE: 1500/28 ms, FA: 90°, ST: 3 mm, NEX: 1, FOV: 150 × 150) for both the open-mouth and closed-mouth positions.

### 2.5 Image evaluation

An experienced radiologist specializing in MRI of the TMJ evaluated all of the scans. Sagittal oblique sequences were utilized to assess the presence of anterior or posterior disc displacement. Both open- and closed-mouth images were analyzed to comprehensively evaluate disc position and morphology.

Condylar degeneration was examined via the T1 sequence and was classified as either a normal condyle or degenerative condyle (Fig. 1) [17].

**Fig. 1.** T1-weighted images showing the condylar morphologies. Arrows show (a) Normal condyle, (b) degenerative condyle respectively.

The position of the articular disc was assessed using sagittal oblique proton density weighted (PDW) images in the closed-mouth position. The disc was classified as normal when the posterior band was located between the 11:00 and 12:00 positions relative to the condyle. A position below 11:00 was considered to be indicative of anterior disc displacement. In cases where the diagnosis was uncertain, the angle between the posterior band of the disc and a line perpendicular to the condylar head was measured; displacement was indicated when this angle exceeded 30°. The presence of disc reduction was evaluated using PDW images obtained in the open-mouth position.

Based on these criteria, the articular disc was categorized as normal, DDWR or DDWOR (Fig. 2) [10].

**Fig. 2.** PDW MRI images showing normal disc position and anterior DDWR and DDWOR. Arrows show discs on all images. (a,b) Closed-mouth (a) and open-mouth (b) PDW images. A normal TMJ with a normal disc was observed. (c,d) Closed-mouth (c) and open-mouth (d) PDW images. Anterior DDWR was observed. (e,f) Closed-mouth (e) and open-mouth (f) PDW images. Anterior DDWOR was observed.

The presence of joint effusion was evaluated using short tau inversion recovery (STIR) images (Fig. 3) [18].

**Fig. 3.** STIR images showing joint effusion. Arrows show the effusion in the upper joint space. (a) No effusion. (b,c) presence of effusion.

Additionally, disc morphology was assessed based on its shape and thickness. The disc was classified as normal (biconcave) or deformed (any shape deviating from the biconcave form) (Fig. 4) [25].

**Fig. 4.** PDW images showing disc morphology. Arrows show the show the discs morphologies. (a) Normal biconcave shape of a TMJ disc. (b) abnormal folded shape of a TMJ disc. (c) abnormal convex shape of a TMJ disc. (d) abnormal flattened shape of a TMJ disc.

### 2.6 Statistical analysis

Descriptive statistics are reported as the mean ± standard deviation, median, minimum, and maximum values for continuous variables and as frequencies and percentages for categorical variables.

Group comparisons of nominal variables (cross-tabulations) were performed using the chi-square test and Fisher’s exact test.

All of the statistical analyses were conducted using IBM SPSS Version 20 (Chicago, IL, USA), and a *p* value < 0.05 was considered to be statistically significant.

## 3. Results

A total of 54 patients were included in the study, with a mean age of 29.63 ± 3.90 years (range: 15–66 years). The majority of the patients were female (88.9%, n = 48). The mean maximal mouth opening distance was 48.00 ± 8.21 mm, and the median duration of the symptoms was 1 year (0.08–10 years). The demographic and clinical characteristics of the patients are presented in Table 1.

**Table 1.** The demographic and clinical characteristics of the patients (n = 54).

Parameter		Value
Age (yr) Median (Min–Max)		25 (15–66)
Mouth opening (mm) Mean ± SD		48.00 ± 8.21
Duration of symptoms (yr) Median (Min–Max)		1 (0.08–10)
Gender n (%)		
	Female	48 (88.9)
	Male	6 (11.1)

*SD: Standard Deviation; Min: Minimum; Max: Maximum.*

### 3.1 Clinical findings

Joint pain was reported in 44.4% (n = 24) of the patients on the right side and in 40.7% (n = 22) of the patients on the left side.

Joint sounds were detected in 44.4% (n = 24) of the patients on both the right and left sides.

Mouth opening limitations were observed in 14.8% (n = 8) of the patients, whereas joint locking was present in 35.2% (n = 19) of the patients.

Deviation or deflection during mouth opening was noted in 55.6% (n = 30) of the patients.

The clinical findings of the patients are provided in Table 2.

**Table 2.** Clinical findings of the patients.

	Right TMJ n (%)	Left TMJ n (%)
Joint pain	24 (44.4)	22 (40.7)
Joint sounds	24 (44.4)	24 (44.4)
Limited mouth opening	8 (14.8)	8 (14.8)
Locking	19 (35.2)	19 (35.2)
Deviation or Deflection	30 (55.6)	30 (55.6)

*TMJ: temporomandibular joint.*

### 3.2 MRI findings

Condylar degeneration was identified in 53.7% (n = 29) of the patients on the right side and in 50.0% (n = 27) of the patients on the left side.

In the right joint, DDWR was observed at a rate of 24.1% (n = 13), whereas DDWOR was observed at a rate of 27.8% (n = 15).

In the left joint, DDWR was observed at a rate of 25.9% (n = 14), whereas DDWOR was observed at a rate of 25.9% (n = 14).

Joint effusion was present in 40.7% (n = 22) of the patients on the right side and in 38.9% (n = 21) of the patients on the left side.

Disc deformities were observed in 33.3% (n = 18) of the patients on the right side and in 29.6% (n = 16) of the patients on the left side.

The MRI findings of the patients are presented in Table 3.

**Table 3.** MRI findings of patients.

	Right TMJ n (%)	Left TMJ n (%)
Condylar degeneration	29 (53.7)	27 (50.0)
Position of the disc	28 (51.9)	28 (51.9)
DDWR	13 (24.1)	14 (25.9)
DDWOR	15 (27.8)	14 (25.9)
Effusion	22 (40.7)	21 (38.9)
Disc deformity	18 (33.3)	16 (29.6)

*TMJ: temporomandibular joint; DDWR: disc displacement with reduction; DDWOR: disc displacement without reduction.*

### 3.3 Comparison of MRI findings and clinical findings

#### 3.3.1 Condylar degeneration and clinical findings

A significant difference was observed in the rate of sound occurrence in the right joint between patients with and without right condylar degeneration (*p* < 0.05). Moreover, the rate of sound occurrence in the right joint was observed to be lower in patients with the right condylar degeneration.

No significant relationships were observed between condylar degeneration and joint pain, deviation/deflection, or locking (*p* > 0.05).

No statistically significant difference was observed between patients with and without condylar degeneration in the left joint regarding pain, joint sounds, limitations in mouth opening, locking, deviation, or deflection (*p* > 0.05).

A comparison of the clinical findings between patients with and without condylar degeneration in the right and left condyles on MRI is presented in Table 4.

**Table 4.** Comparison of clinical findings between patients with and without condylar degeneration in the right and left condyles on MRI.

	No Right Condylar Degeneration		Right Condylar Degeneration Present		*p* value
	n	%	n	%	
Pain in right joint	11	44.0	13	44.8	0.951^c^
Mouth opening limitation	3	12.0	5	17.2	0.711^c^
Sound in right joint	15	60.0	9	31.0	0.033^c^
Deviation/Deflection	12	48.0	18	62.1	0.300^c^
Locking	6	24.0	13	44.8	0.110^c^
	No Left Condylar Degeneration		Left Condylar Degeneration Present		*p* value
	n	%	n	%	
Pain in left joint	11	40.7	11	40.7	1.000^c^
Mouth opening limitation	3	11.1	5	18.5	0.704^c^
Sound in left joint	13	48.1	11	40.7	0.584^c^
Deviation/Deflection	13	48.1	17	63.0	0.273^c^
Locking	8	29.6	11	40.7	0.393^c^

*c: Chi-square test/Fisher’s exact test.*

#### 3.3.2 Disk position disorders and clinical findings

MRI revealed a significant difference in the incidence of pain in the right joint among patients with normal, DDWR and DDWOR classifications (*p* < 0.05). The prevalence of pain in patients with DDWR and DDWOR was greater than that in patients with a normal disc position (*p* < 0.05 for both). However, there was no significant difference observed in the prevalence of pain between patients with DDWR and those with DDWOR.

On MR images, a significant difference was observed in the prevalence of sound occurrences in the right joint among patients with normal, DDWR and DDWOR classifications (*p* < 0.05). Additionally, a significant difference was observed in the prevalence of sound occurrences between patients with DDWR and those with DDWOR (*p* < 0.01). The prevalence of sound occurrences in patients with DDWR was greater than that in patients with DDWOR.

In the MR images, no significant differences were observed in the prevalence of mouth opening limitations, deviations/deflections, or locking among patients with normal, DDWR and DDWOR classifications (*p* > 0.05).

On MRI, there were no statistically significant differences observed in pain, limitations in mouth opening, sounds on the left side, deviations/deflections, or locking rates in the left joint among patients with normal, DDWR and DDWOR classifications of the left joint (*p* > 0.05).

A comparison of the clinical findings among patients with normal, DDWR or DDWOR classifications in the right and left joints on MRI is presented in Table 5.

**Table 5.** Comparison of clinical findings among patients with normal, DDWR or DDWOR classification in the right and left joints on MRI.

	Normal in the right side		DDWR in the right side		DDWOR in the right side		*p* value
	n	%	n	%	n	%	
Pain in right joint	7	26.9	8	61.5	9	60.0	0.044^c^
Mouth opening limitation	2	7.7	1	7.7	5	33.3	0.093^c^
Sound in right joint	12	46.2	9	69.2	3	20.0	0.032^c^
Deviation/Deflection	16	61.5	7	53.8	7	46.7	0.647^c^
Locking	6	23.1	6	46.2	7	46.7	0.199^c^
	Normal in the left side		DDWR in the left side		DDWOR in the left side		*p* value
	n	%	n	%	n	%	
Pain in left joint	10	38.5	6	42.9	6	42.9	0.947^c^
Mouth opening limitation	3	11.5	2	14.3	3	21.4	0.881^c^
Sound in left joint	11	42.3	9	64.3	4	28.6	0.157^c^
Deviation/Deflection	13	50.0	7	50.0	10	71.4	0.381^c^
Locking	5	12.9	7	50.0	7	50.0	0.055^c^

*c: Chi-square test/Fisher’s exact test; DDWR: disc displacement with reduction; DDWOR: disc displacement without reduction.*

Additionally, a significant relationship was observed regarding condylar degeneration in patients with DDWR or DDWOR on MRI (*p* < 0.001). There was a significant difference observed in effusion rates between the DDWR and DDWOR classifications, with the disk position being normal in the MR images (*p* < 0.001). The rate of effusion was observed to be higher in those with DDWR and DDWOR than in those with a normal disk position. No significant difference was observed in the effusion rates between the DDWR and DDWOR classifications (*p* > 0.05).

#### 3.3.3 Effusion and clinical findings

No significant differences were detected between effusion and pain, sounds, limitations in mouth opening, locking, deviations, or deflections in the right and left joints (*p* > 0.05).

A comparison of the clinical findings of patients with and without effusion in the right and left joints on MRI is provided in Table 6.

**Table 6.** Comparison of the clinical findings of patients with and without effusion in the right and left joints on MRI.

	No effusion on the right side		Right effusion present		*p* value
	n	%	n	%	
Pain in right joint	11	34.4	13	59.1	0.073^c^
Mouth opening limitation	3	9.4	5	22.7	0.248^c^
Sound in right joint	14	43.8	10	45.5	0.901^c^
Deviation/Deflection	16	50.0	14	63.6	0.322^c^
Locking	10	31.2	9	40.9	0.465^c^
Pain in left joint	12	36.4	10	47.6	0.412^c^
Mouth opening limitation	4	12.1	4	19.0	0.697^c^
Sound in right joint	17	51.5	7	33.3	0.190^c^
Deviation/Deflection	16	48.5	14	66.7	0.190^c^
Locking	9	27.3	10	47.6	0.127^c^

*c: Chi-square test/Fisher’s exact test.*

Additionally, a significant difference was observed in the effusion rates between joints with and without condylar degeneration on MRI (*p* < 0.05). The presence of effusion was more frequently observed in joints with condylar degeneration on MRI.

#### 3.3.4 Disc deformity and clinical findings

Patients with deformed left disc shapes demonstrated a significantly reduced degree of mouth opening (*p* < 0.05).

No significant differences were observed between disc shape deformities in the right and left joints and joint pain, sounds, restricted maximum mouth opening, locking, deviations or deflections (*p* > 0.05).

A comparison of the clinical findings of patients with normal and distorted disc shapes on the right and left sides on MR images is shown in Table 7.

**Table 7.** Comparison of the effusion and disc position rates in MRI findings between joints with normal disc shapes and joints with deterioration.

		Disc shape normal		Disc shape is deformed		*p* value
		n	%	n	%	
Effusion		20	27.0	23	67.6	<0.001^c^
Position of disc						
	Normal	50	67.6	2	5.9	<0.001^c^
	DDWR	17	23.0	10	29.4	
	DDWOR	7	9.5	22	64.7	

*c: Chi-square test/Fisher’s exact test; DDWR: disc displacement with reduction; DDWOR: disc displacement without reduction.*

Additionally, in the MRI findings, a difference was detected between the rates of disc deformity in the MRI findings of the joints with no condyle degeneration and those of joints with degeneration (*p* < 0.05). The rate of disc deformation was observed to be greater in patients with condylar degeneration on MRI.

## 4. Discussion

TMDs exhibit characteristic clinical signs and symptoms, including pain in the TMJ, facial pain, joint noises, mouth opening limitations, locking, deviations or deflections. MRI of the TMJ has been accepted as the best method for diagnosing the status of the TMJ [20, 22]. In this study, the relationships between various conditions (such as condylar degeneration, disc displacement, the presence of effusion and disc deformity detected on MRI) and clinical findings were evaluated. MRI is essential for detecting early signs of TMD so that appropriate treatments may be provided to prevent the irreversible phase of degenerative changes that result in a dysfunctional TMJ.

Degenerative bone changes in the TMJ include osteophytes, erosion, avascular necrosis, subchondral cysts, and intra-articular loose bodies present in the condyle (rather than in the articular eminence) [3, 4].

Bertram *et al*. [5] reported a significant relationship between pain and osteoarthritis in the TMJ in their study involving 131 participants (*p* = 0.013). Similarly, Emshoff *et al*. [7] conducted a study involving 112 participants and reported a positive association between pain and osteoarthritis (χ^2^ = 15.868; *df* = 1; *p* < 0.001). Furthermore, in another study with 578 participants, Emshoff *et al*. [6] reported a relationship between pain and osteoarthritis in the TMJ (Chi-square = 8.146; *p* = 0.004; *df* = 1).

Honda *et al*. [8] classified condylar degeneration into the following two groups: pathological and adaptive bone changes. Using electrovibratography, they reported that high-frequency joint sounds were significantly more frequent and pronounced in pathological bone changes than in adaptive bone changes (*p* < 0.05).

In another study involving 796 participants, Vogl *et al*. [10] reported a significant correlation between condylar degeneration and restricted mouth opening (*p* < 0.005). They reported that the average distance of mouth opening was 37 mm in individuals with normal condyles, whereas it was 34 mm in those with degenerative condyles.

Matsubara *et al*. [12] reported that there were significant associations between joint pain and the presence of osteophytes in the condyle (*p* = 0.02), as well as combined condylar degeneration (*p* = 0.01). They also reported a significant relationship between flattened condyles and joint sounds (*p* = 0.01) and between flattened condyles and restricted mouth opening (*p* = 0.05).

In our study, no significant relationships were observed between osteoarthritic condyles and joint pain, joint sounds, or restricted mouth opening (*p* > 0.05). However, the presence of joint sounds was greater in joints without degeneration in the right condyle than in those with degeneration (*p* = 0.03). This finding suggests that disc displacement may be a contributing factor to this scenario.

TMDs can manifest due to irregular or degenerated intra-articular components. The most common cause of internal derangement involves an imbalance between the condyle, temporal bone, and articular disc [13].

Emshoff *et al*. [6] demonstrated a significant relationship between internal derangements of the temporomandibular joint and pain (*p* = 0.002). In a separate study, Hosgor reported that patients with disc displacement demonstrated higher VAS scores (*p* < 0.05).

Aligning with these studies, Bertram *et al*. [5] emphasized a significant relationship between internal derangement (*p* < 0.001) and temporomandibular joint disorders, specifically with respect to pain as a clinical symptom and osteoarthritis as an MRI finding.

Vogl *et al*. [10] reported that as the degree of mouth opening decreases, the likelihood of internal disorders increases (*p* < 0.001).

Matsubara *et al*. [12] reported a significant relationship between pain (*p* < 0.001) and sound (*p* < 0.001) in joints in the presence of DDWOR. They also reported that there was a significant relationship between DDWR and DDWOR, as well as between DDWR and limitations in mouth opening (*p* < 0.001).

In their study, Yasan *et al*. [14] reported that the rates of pain (*p* = 0.007), limitations in mouth opening (*p* < 0.001) and the percentage of 3rd degree effusion (*p* < 0.001) in the presence of DDWOR were greater than those in other groups.

In our study, although there was a significant relationship observed between DDWR and DDWOR in the right TMJ and clinical findings such as pain (*p* = 0.044) and sound (*p* = 0.032), no relationship was observed with limitations in mouth opening (*p* > 0.05). Additionally, internal derangements in the TMJ were observed to be associated with condylar degeneration and effusion (*p* < 0.001).

TMJ effusion is an indicator of intra-articular pathology, and it is hypothesized that this type of effusion should be present in painful joints. However, the relationship between radiological evidence of effusion in the TMJ and clinical symptoms (such as pain) remains unclear [15].

In a study involving 379 patients, Westesson and Brooks [19] reported a strong relationship between MRI-detected joint effusion and pain (*p* < 0.001). In a study conducted by Hosgor [15], effusion in the joints of 120 patients was graded, and patients exhibiting significant effusion had higher VAS scores than did those without effusion (*p* < 0.05).

Vogl *et al*. [10] reported a strong correlation between mouth opening and joint effusion (*p* < 0.05). Moreover, the average mouth opening distance was observed to be 39 mm in the absence of effusion, whereas it was 36 mm and 34 mm in the presence of effusion.

Matsubara *et al*. [12] reported that the presence of Grade 2 and Grade 3 effusion in the TMJ was associated with joint pain (Grade 2: *p* = 0.01; Grade 3: *p* < 0.001) and sound (Grade 2: *p* < 0.001; Grade 3: *p* < 0.001) but did not affect mouth opening.

Unlike in other studies, our study demonstrated that joint effusion had no effect on clinical findings such as pain, sound, or mouth opening (*p* > 0.05). However, the presence of effusion in the TMJ was observed to be associated with condylar degeneration (*p* = 0.025).

The deformity of disc shape has been shown to be associated with internal derangement of the TMJ and degenerative changes in the condyles [12, 14].

Yasan *et al*. [14] reported higher percentages of different disc configurations in patients with varying disc positions. In patients with a normal disc position, the percentage of biconcave disc configurations was greater (30.6% *vs.* 3.7% and 0.0%, respectively). In patients with DDWR, the percentage of flattened disc configurations was greater (79.4% *vs.* 52.7%). Moreover, in patients with DDWOR, the percentages of convex disc configurations (14.9% *vs.* 0.0%) and folded disc configurations (32.4% *vs.* 0.0% and 10.3%) were also greater (*p* < 0.001).

Matsubara *et al*. [12] reported a strong positive correlation between biconcave disc position and normal disc position (*r* = 0.706, *p* < 0.01). They also reported a moderate positive correlation between a folded disc configuration and DDWOR (*r* = 0.467, *p* < 0.01), a weak positive correlation between a flattened disc configuration and DDWR (*r* = 0.320, *p* < 0.01), and a weak negative correlation between DDWOR and the normal disc configuration (*r* = −0.323, *p* < 0.01).

This study revealed that there was a relationship between disc shape alteration (configuration) and condylar degeneration (*p* < 0.05), joint effusion (*p* < 0.001), and disc position (*p* < 0.001). In both DDWR and DDWOR classifications, disc configuration was observed to be altered in cases of condylar degeneration and the presence of joint effusion.

The main limitation of this study is the insufficient number of patients, which prevents the classifications of effusion grade, condylar degeneration type, and disc shape. Another limitation of the study includes the use of the MRI technique. Images of the joints were only obtained in the sagittal oblique plane. For this reason, disc displacement was unidirectionally evaluated (in the anterior-posterior direction). To view the medial or lateral displacement of the disc, images in the coronal and axial planes should be obtained in addition to images in the sagittal plane. The final limitation of this study is that the data were collected between October 2019 and March 2020. This time period may not fully reflect changes that may have occurred over time in clinical practice, treatment protocols or diagnostic methods.

## 5. Conclusions

In this study, the relationship between magnetic resonance imaging (MRI) findings and clinical symptoms in temporomandibular joint disorders (TMDs) is emphasized. Displacement of the disc with and without reduction was significantly associated with pain and joint sounds, whereas joint effusion was not clearly correlated with clinical symptoms. Condylar degeneration was commonly observed but had a limited clinical impact (except for a reduction in joint sounds). This study also revealed that disc deformity was associated with condylar degeneration and joint effusion. These findings highlight the importance of MRI in the accurate diagnosis and treatment planning of TMDs.
